# Supplementary material for: Gestational Interrelationships among Gut–Metabolism–Transcriptome in Regulating Early Embryo Implantation and Placental Development in Mice
Source: Microorganisms. 2024 Sep 17;12(9):1902. doi: 10.3390/microorganisms12091902 (PMC11434064; doi:10.3390/microorganisms12091902)
Supplement: Supplementary file 1 [file microorganisms-12-01902-s001.zip › Supplementary materials.pdf]

Gestational Interrelationships among Gut–Metabolism–Transcriptome in Regulating Early Embryo Implantation and Placental Development in Mice

Shuai Lin *,†, Yuqi Liang †, Jingqi Geng, Yunfei Yan, Ruipei Ding and Maozhang He *

School of Basic Medical Sciences, Anhui Medical University, Hefei 230032, China; 18907757553@163.com (Y.L.); gengjq1013@163.com (J.G.); 18236850769@163.com (Y.Y.); 2113100010@stu.ahmu.edu.cn (R.D.)

* Correspondence: shuailin@ahmu.edu.cn (S.L.); hmz91@ahmu.edu.cn (M.H.)

† These authors contributed equally to this work.

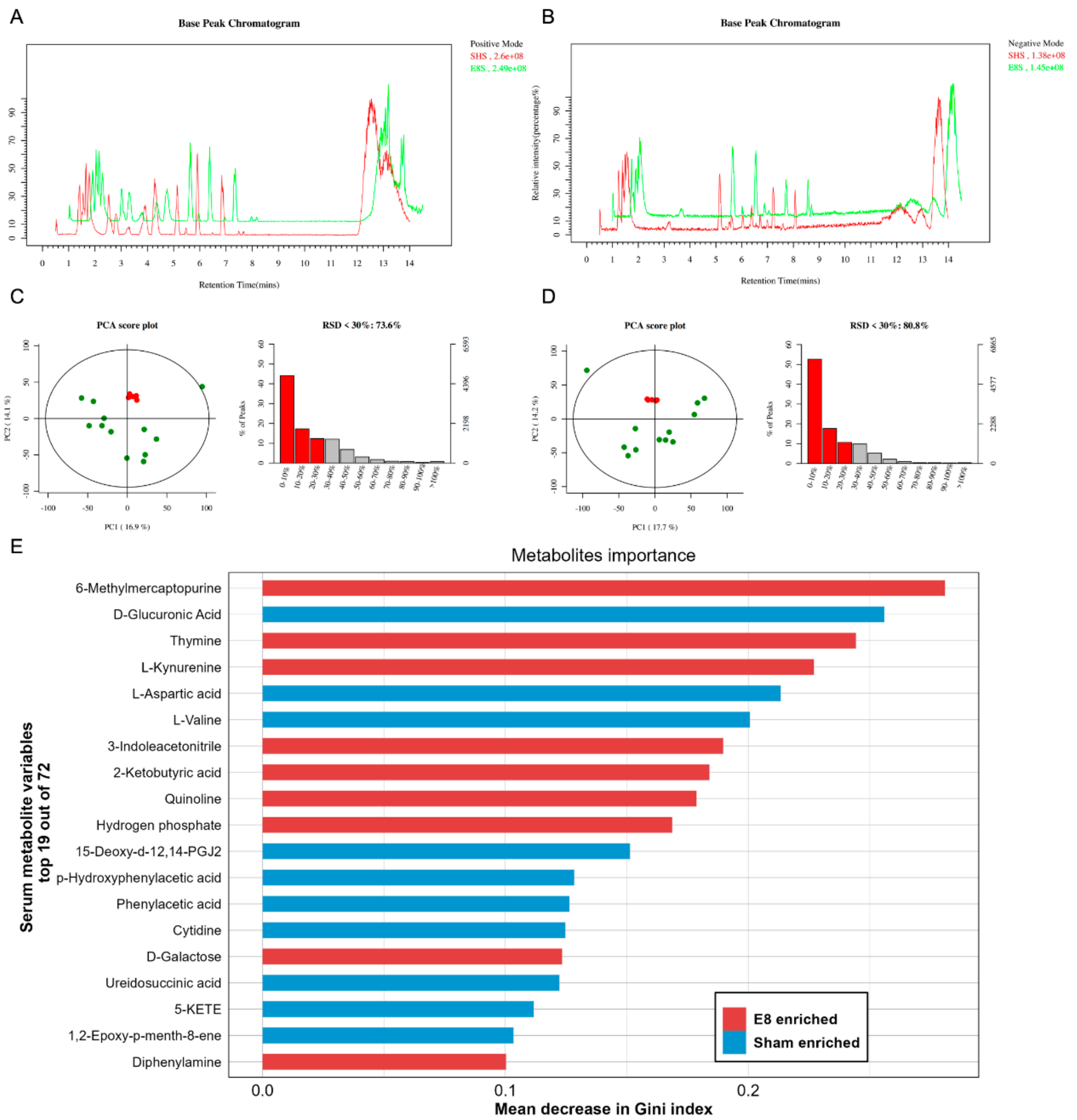

Figure S1. The quality assessment of serum metabolomics. (A-B) Typical Base Peak Chromatogram of the ESI+ (A) and ESI- (B) modes. (C-D) Score plots of principal component analysis based on the ESI+ and ESI− modes. Red solid circle represented the QC samples (left panel). CV distribution of peaks in combinational dataset of ESI+ and ESI- modes. The columns represent the number of peaks and accumulative percentage of peaks in corresponding CV interval, respectively (right panel). (E) Variable importance plot of top 19 serum metabolites (y-axis) ranked by the contribution to mean decrease accuracy of Gini coefficient (x-axis) in the random forest model for discerning group difference.

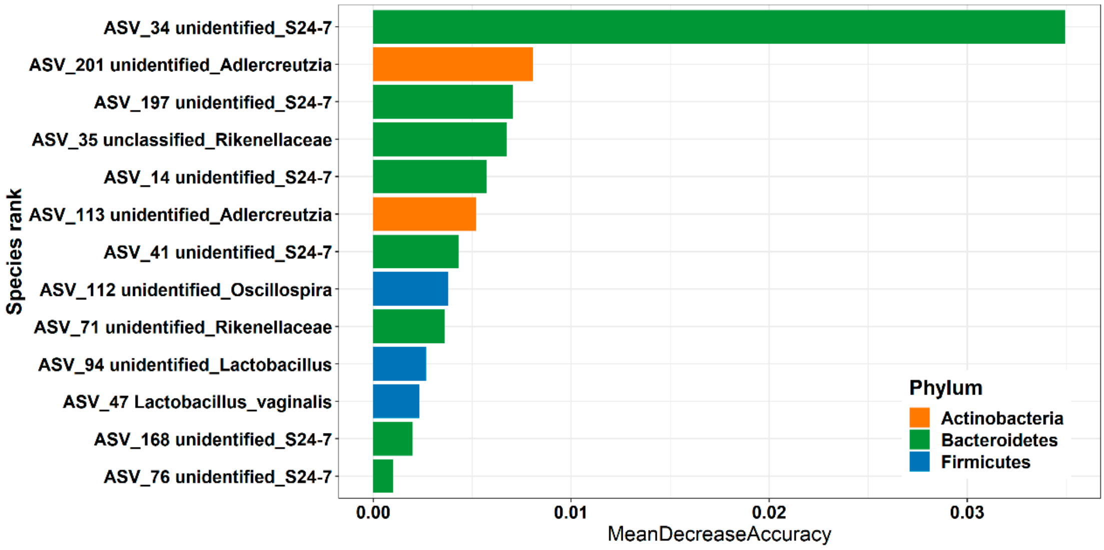

Figure S2. The top 13 biomarkers of ASVs that could discriminate the samples from E8 and Sham by Random Forest model. Biomarker ASVs were ranked in descending order of importance to the accuracy of the model.

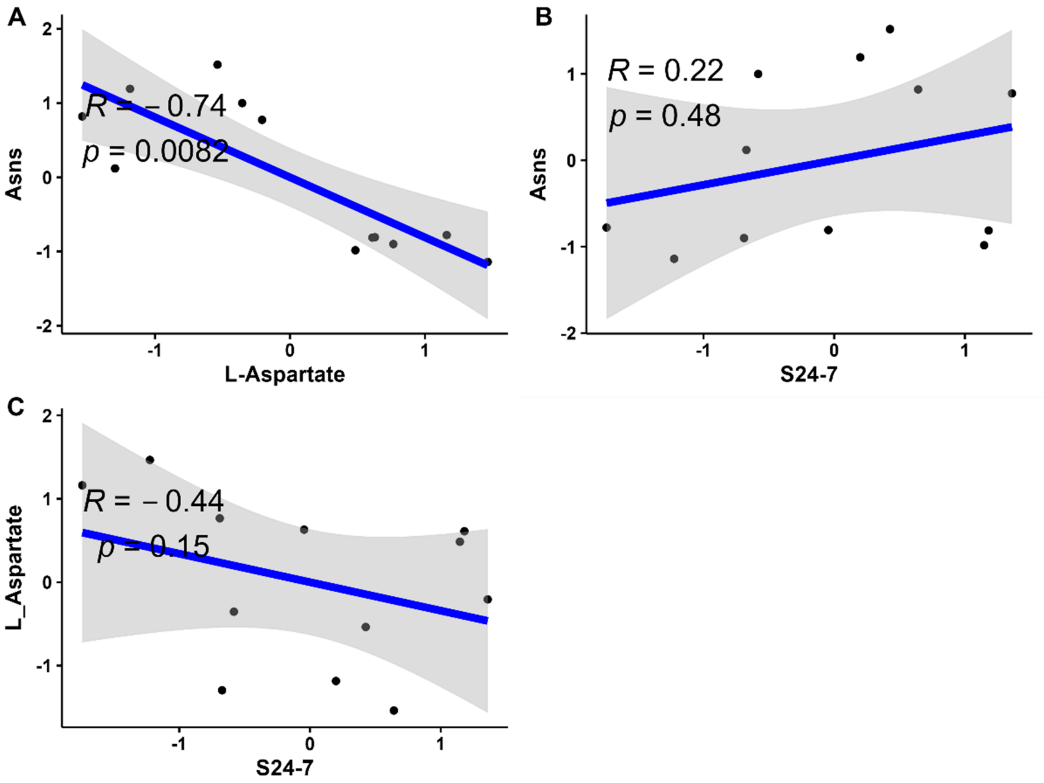

Figure S3. Scatter plots representing the negative relationship between the Asns gene and L-Aspartate metabolite (A), while showed positive correlation between Asns and microbial S24-7 (B), L-Aspartate negative correlated with S24-7 (C).

Supplementary Table Legends

Table S1. The information and intensity of annotated metabolites.

Table S2. The 73 metabolites that significant differential between E8 and Sham mice.

Table S3. A total of 22 significant distinct ASVs that between E8 and Sham mice.

Table S4. The differential abundant genes between E8 and Sham mice.

Table S5. KEGG pathways that enriched by the differential genes.

Table S6. The enriched GO items based on differential genes.

Table S7. The associations between the differential gut microbiota and serum metabolites by spearman correlations analysis.

Table S8. The module assignment of differential metabolites by WGCNA analysis.
